# Serpent/dGATAb regulates *Laminin B1* and *Laminin B2* expression during *Drosophila* embryogenesis

**DOI:** 10.1038/s41598-019-52210-9

**Published:** 2019-11-04

**Authors:** Uwe Töpfer, Maik C. Bischoff, Marek Bartkuhn, Anne Holz

**Affiliations:** 1 Justus-Liebig-Universitaet Giessen, Institut fuer Allgemeine und Spezielle Zoologie, Allgemeine Zoologie und Entwicklungsbiologie, Stephanstrasse 24, 35390 Giessen, Germany; 2 Present Address: Technische Universitaet Dresden, Institut fuer Genetik, Zellescher Weg 20b, 01217 Dresden, Germany; 3 Present Address: Philipps-Universitaet Marburg, AG Entwicklungsbiologie der Tiere, Karl-von-Frisch-Strasse 8, 35043 Marburg, Germany; 4 Justus-Liebig-Universitaet Giessen, Institut fuer Genetik, Heinrich-Buff-Ring 58-62, 35392 Giessen, Germany

**Keywords:** Extracellular matrix, Morphogenesis, Gene regulation

## Abstract

Transcriptional regulation of *Laminin* expression during embryogenesis is a key step required for proper ECM assembly. We show, that in *Drosophila* the *Laminin B1* and *Laminin B2* genes share expression patterns in mesodermal cells as well as in endodermal and ectodermal gut primordia, yolk and amnioserosa. In the absence of the GATA transcription factor Serpent, the spatial extend of *Laminin* reporter gene expression was strongly limited, indicating that Laminin expression in many tissues depends on Serpent activity. We demonstrate a direct binding of Serpent to the intronic enhancers of *Laminin B1* and *Laminin B2*. In addition, ectopically expressed Serpent activated enhancer elements of *Laminin B1* and *Laminin B2*. Our results reveal Serpent as an important regulator of *Laminin* expression across tissues.

## Introduction

Laminins are heterotrimeric proteins found in the extracellular matrix (ECM) and are major components of all basement membranes (BMs). These proteins self-assemble into a cell-associated network and interact with cell-surface molecules such as integrin receptors and other ECM components. Laminin heterotrimers are composed of three distinct subunits (α, β, and γ) that yield a cross-shaped structure. The *Drosophila* genome encodes two distinct α-subunits (Laminin A, LanA; Laminin Wing blister, LanWb), as well as one β- (Laminin B1, LanB1) and one γ-subunit (Laminin B2, LanB2)^1,2^. Each heterotrimer is formed by initial dimerization of ß- and γ-subunits via disulfide-bonding and subsequent incorporation of α-subunits, followed by the secretion and binding of the final receptor at the cell surface^3–6^. There, Laminins perform several functions in higher organisms, ranging from cell adhesion to migration processes during development^7–9^. Experiments using mammalian cell culture revealed that α-subunits can be secreted independently, whereas the secretion of β/γ proteins needs simultaneous expression of both^10^, indicating a common regulatory mechanism for them. Moreover, loss of LanB1 and LanB2 pointed to a dependency of both proteins for heterotrimeric Laminin-secretion in *Drosophila*^7,8,11^.

While Laminins and their roles in BM self-assembly are well investigated in mammalian cell culture, the regulation of this process is poorly understood. With respect to the single ß-/γ-subunits, *Drosophila* seems to be a suitable model to study Laminin gene regulation *in vivo*, since mammalian genomes contain twelve distinct Laminin subunits^12^. Due to the strong expression of Laminins in *Drosophila* hemocytes and fat body cells, as well as the observation of severe endodermal defects in *Laminin* mutant embryos, we focused our analysis on the main transcriptional regulator of these tissues in *Drosophila*, the GATA-transcription factor Serpent (Srp, dGATAb). Srp is involved in the differentiation of several tissues derived from different germ layers, such as mesoderm derived fat body and blood cells, endodermal midgut primordia, and the amnioserosa^13^. Notably, these are tissues in which the Laminin subunits B1 and B2 also play an important role during morphogenesis^7,8,14^.

## Results

### Reporter gene expression for *LanB1* and *LanB2*

**Figure 1:**
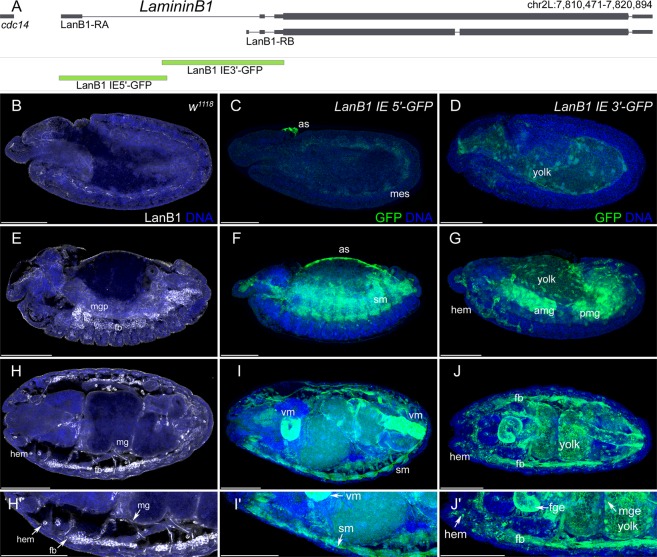
Embryonic expression of *LanB1* reporter gene constructs. (**A**) Schematic representation of the *LanB1* genetic region and the derived reporter constructs. **(B**,**E**,**H**,H′) Protein distribution of LanB1 (white) in *white*^*1118*^ (*w*^*1118*^) embryos. **(C**,**F**,**I**,I′) Reporter gene expression (green) of the *LanB1 IE 5*′*-GFP* construct. **(D**,**G**,**J**,J′) Reporter gene expression (green) of the *LanB1 IE* 3′*-GFP* construct. **(B**–**D)** Embryos at stage 11 (lateral view), **(E–G)** embryos at stage 14 (lateral view), **(H**–**J)** embryos at stage 16 (dorsal view) and (H′-J′) higher magnification of the embryos in (**H**–**J**). **(B-**J′) DNA staining in blue. Abbr.: as: amnioserosa, amp and pmg: anterior and posterior midgut primordia, fb: fat body, fge: foregut epithelium, hem: hemocytes, mes: mesoderm, mge: midgut epithelium, sm: somatic muscles, vm: visceral muscles. Scale Bars = 100 µm.

**Figure 2:**
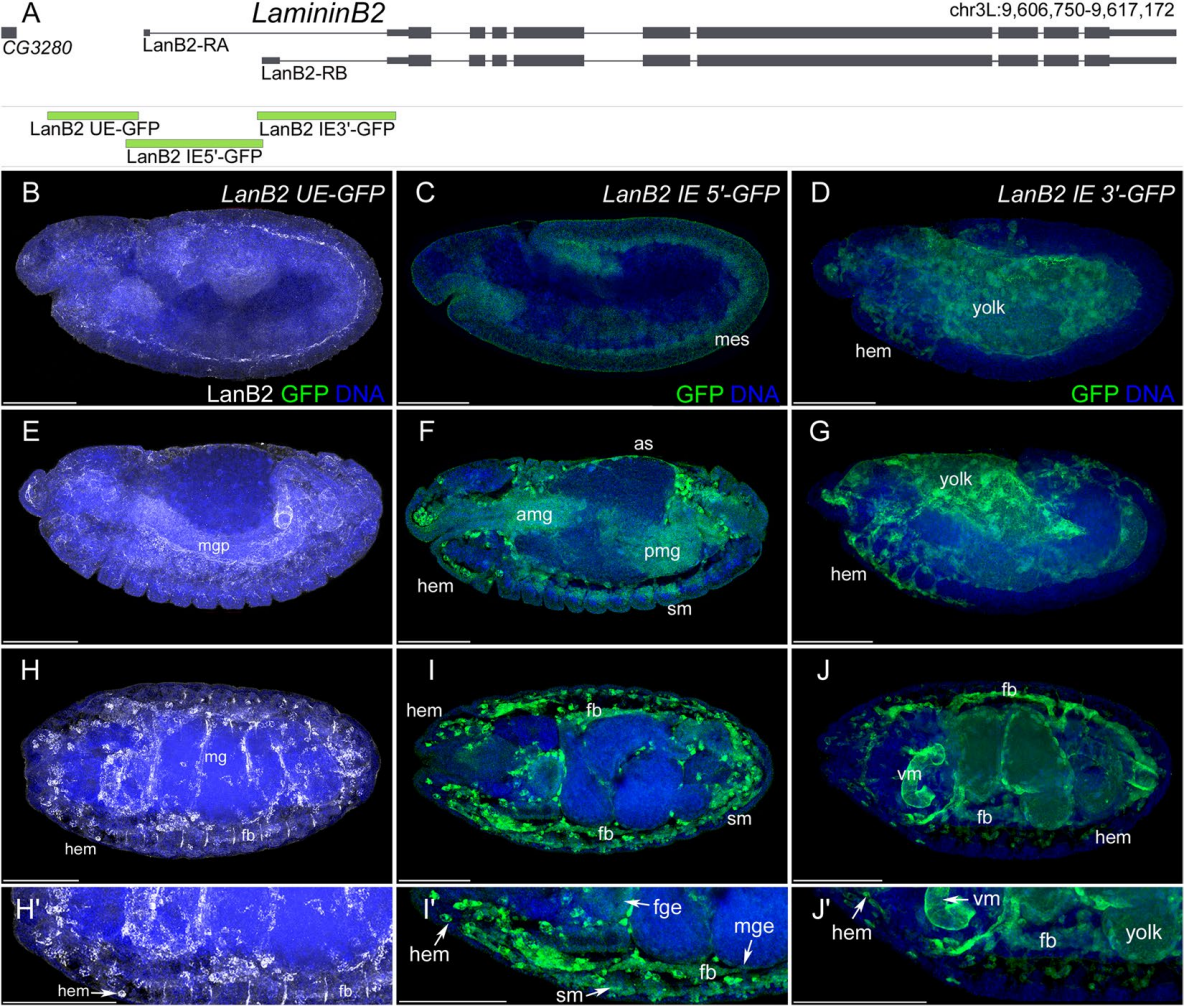
Embryonic expression of *LanB2* reporter gene constructs. (**A**) Schematic representation of the *LanB2* genetic region and the derived reporter constructs. **(B**,**E**,**H**,H′) Reporter gene expression (green) of the *LanB2 UE-GFP* construct and LanB2 protein distribution (white). **(C**,**F**,**I**,I′**)** Reporter gene expression (green) of the *LanB2 IE 5*′*-GFP* construct. **(D**,**G**,**J**,J′) Reporter gene expression (green) of the *LanB2 IE 3*′*-GFP* construct. **(B**–**D)** Embryos at stage 11 (lateral view), **(E**–**G**) embryos at stage 14 (lateral view), **(H**–**J)** embryos at stage 16 (dorsal view) and **(**H′–J′) higher magnification of the embryos in (**H**–**J**). **(B**–J′) DNA staining in blue. Abbr.: as: amnioserosa, amp and pmg: anterior and posterior midgut primordia, fb: fat body, fge: foregut epithelium, hem: hemocytes, mes: mesoderm, mge: midgut epithelium, sm: somatic muscles, vm: visceral muscles. Scale Bars = 100 µm.

During late embryonic stages, most of the Laminin proteins present in the ECM of tissues are synthesized and secreted by hemocytes and the fat body. However, during early embryonic development, Laminin expression is not detected in hemocytes or the fat body, indicating that during this stage Laminin proteins are produced by the tissues themselves (Figs 1 and 2). We selected the *Drosophila* genes *Laminin B1* and *Laminin B2*, encoding the unique Laminin ß- and γ-subunit, owing to their postulated co-expression^7,8,15,16^, to characterize gene expression of early embryonic tissues and late embryonic secretion. Both genes show small upstream (upstream enhancer, UE) and large intronic (intronic enhancer, IE) enhancers. Bioinformatic analysis of the large first introns revealed several conserved regions in these enhancers, indicating the presence of cis-regulatory modules (CRMs). Only *LanB2* displayed an additional small conserved region in the corresponding UE. Therefore, we generated reporter constructs by fusing the derived CRMs of both *Laminin* genes to GFP, analyzed the derived tissue-specific expression and compared it to the described mRNA and protein distribution (Supplementary Table S1 and Figs 1 and 2).

The *IE 5*′ constructs of both Laminins revealed expression in the early mesoderm as well as in the amnioserosa (*LanB1 IE 5*′ in Fig. 1C,F and *LanB2 IE 5*′ in Fig. 2C,F, amnioserosa not visible in 2 C). At the end of embryogenesis, expression was found in the mesoderm derived somatic muscles (*LanB1 IE 5*′ in Fig. 1I,I′ and *LanB2 IE 5*′ in Fig. 2I,I′) and the visceral muscles (*LanB1 IE 5*′ in Fig. 1I,I′ and *LanB2 IE 3*′ in Fig. 2J,J′). Additional expression in endodermal midgut primordia was displayed for the *LanB1 IE 3*′ (Fig. 1G) as well as *LanB2 IE 5*′ (Fig. 2F) construct.

Laminin reporter gene expression in hemocytes and fat body cells could be detected from early differentiation and was observed for the *LanB2 IE 5*′ construct (*LanB2 IE 5*′ in Fig. 2F,I,I′) as well as in both 3′ constructs (*LanB1 IE 3*′ in Fig. 1G,J,J′ and *LanB2 IE 3*′ in Fig. 2G,J,J′). Both *Lan IE 3*′ constructs showed further expression in some cells of the peripheral nervous system, in the dorsal median cells (both not shown) and the yolk (*LanB1 IE 3*′ in Fig. 1D,G,J,J′ and *LanB2 IE 3*′ in Fig. 2D,G,J,J′). The *LanB1 IE 3*′ construct displayed additional expression in salivary glands and Malpighian tubules (both not shown). Although the *LanB2 UE* region contains a small conserved region (Supplementary Fig. S2), the analysis revealed no embryonic reporter gene expression, indicating no CRMs for embryonic expression in this UE. In summary, the reporter constructs reflect the complete known embryonic expression of *LanB1* and *LanB2* (Supplementary Table S1), so that all CRMs promoting embryonic expression should also be included.

The comparative Laminin B1 and B2 protein distribution appeared initially in a layer between the mesoderm and ectoderm of embryos at the fully elongated germ band stage and was continued in the somatic and visceral mesoderm as well as in the endodermal midgut primordia. At the end of embryogenesis, LanB1 and LanB2 covered most tissues and were strongly secreted by fat body and blood cells (LanB1 in Fig. 1H,H′ and LanB2 in Fig. 2H,H′^7,8^). In conclusion, every tissue in which LanB1 and LanB2 could be detected at the end of embryogenesis seemed to express Laminin itself for its own initial BM assembly.

### *In silico* prediction of putative Srp-binding sites in *LanB1* and *LanB2*

In order to identify transcriptional regulatory mechanisms, we searched for potential regulators controlling *LanB1* and *LanB2* expression using transcription factor binding profile databases^17,18^. Conservation scores (PhastCons datasets of 14 insect species)^19^ were used to identify and eliminate the false positive transcription factor binding sites (TFBSs) enriched in the non-coding regions, based on the assumption that binding sites essential for Laminin expression are strongly conserved across insect phylogeny. We found an overrepresentation of potential binding sites for Srp^20,21^ in the intronic enhancers (IE) of *LanB1* and *LanB2*, and one additional hit in the upstream enhancer (UE) of *LanB2*. These binding sites showed strong conservation scores and were located partially in conserved regions, but also at loci, where only the binding sites displayed peaks of conservation (Supplementary Figs S1 and S2).

### *LanB1* and *LanB2* reporter gene expression in *srp* mutant background and upon tissue-specific *srp* knockdown

**Figure 3:**
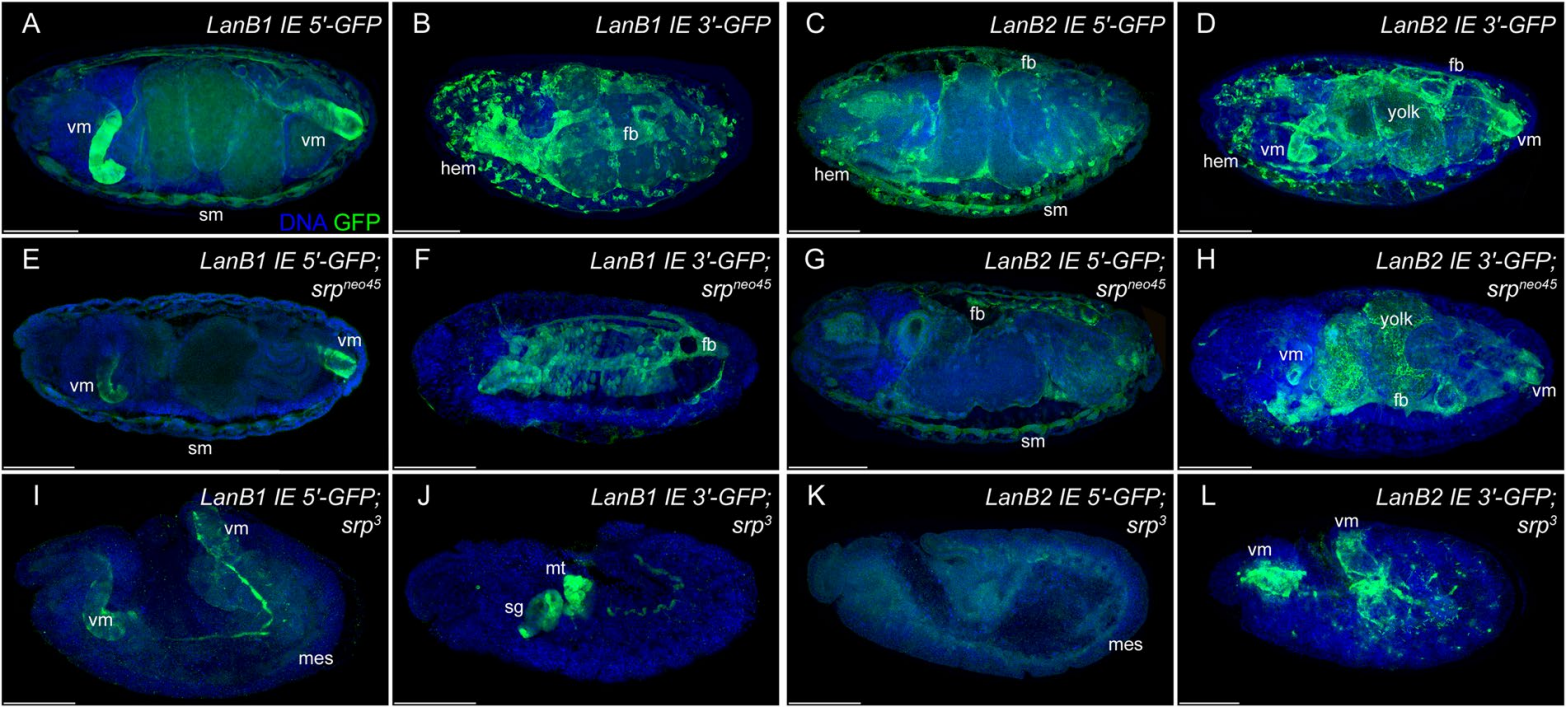
Reporter gene expression in *srp* mutant background. Reporter gene expression of *LanB1* and *LanB2* intronic enhancer regions **(A**–**D)** displayed in the genetic background of hypomorphic (*srp*^*neo45*^) **(E**–**H)** and amorphic (*srp*^*3*^) **(I**–**L)** *srp* alleles. Abbr.: fb: fat body, fg: foregut, hem: hemocytes, hg: hindgut, mes: mesoderm, mt: Malpighian tubules, sg: salivary glands, sm: somatic muscles, vm: visceral muscles. Scale bars = 100 µm.

To test whether expression of *LanB1* and *LanB2* depends on the GATA-factor Serpent (Srp), we analyzed *Laminin* reporter gene expression in hypomorphic *srp*^*neo45*^ mutant embryos, in which only the differentiation of hemocytes is affected, and in *srp*^3^ null mutant embryos^13,22^ (Fig. 3).

In *srp*^*neo45*^ mutants, hemocytes were missing and within reporter gene expression of hemocytes, whereas expression in all other tissues was still observed (Fig. 3E–H). In contrast, in *srp*^3^ embryos the fat body and the amnioserosa remain undifferentiated and the hemocytes as well as endoderm primordia were missing^13,23–26^. Only residual reporter gene expression could be detected in the progenitor cells of the visceral and somatic mesoderm (Fig. 3I,K,L), in some cells of the PNS (Fig. 3L), and in the rudiments of salivary glands and Malpighian tubules (Fig. 3J). This remaining reporter gene expression in *srp* mutant embryos revealed additional *srp*-independent *Laminin* regulation.

### Srp binds directly to the intronic enhancers of *LanB1* and *LanB2*

**Figure 4:**
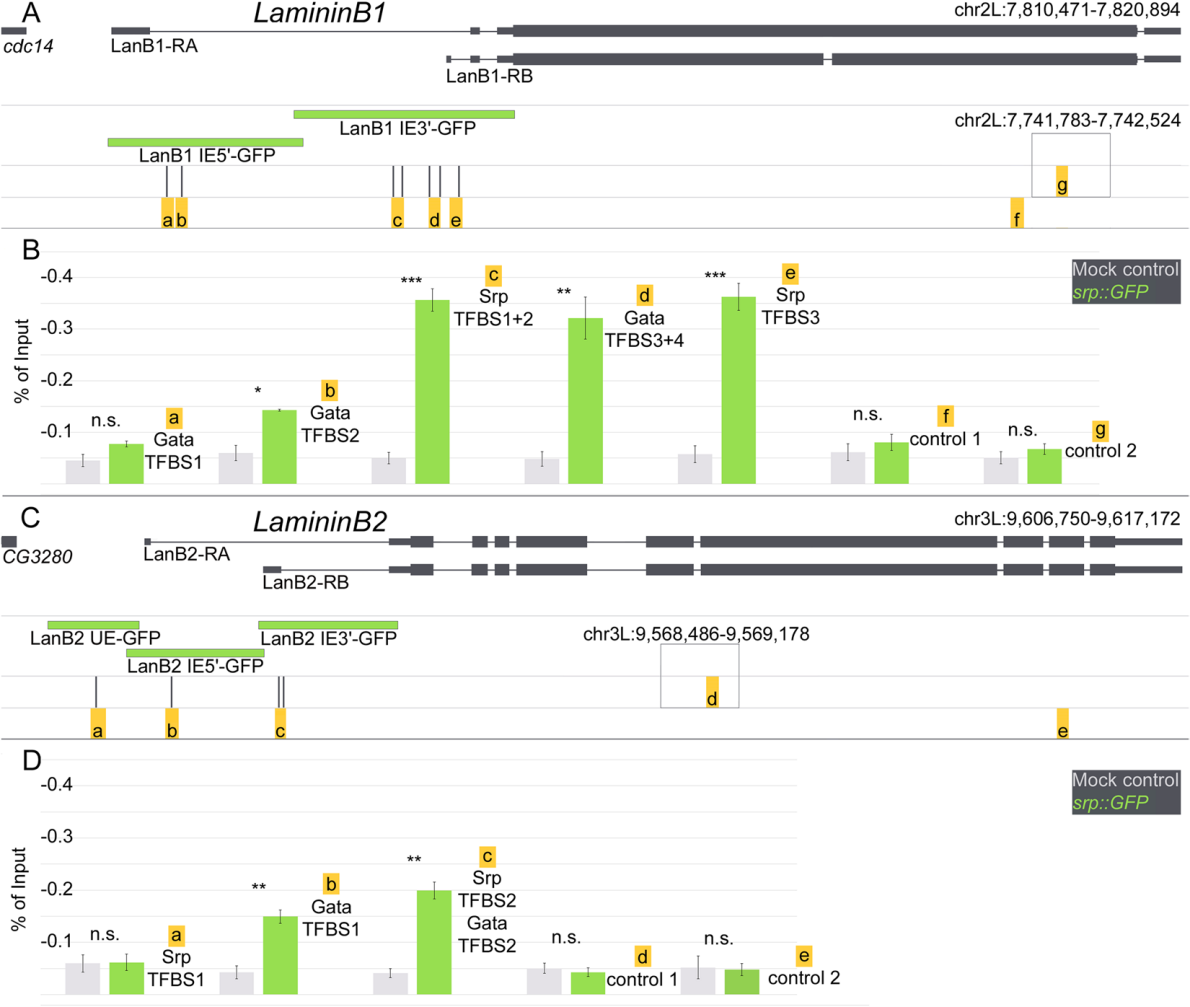
ChIP-qPCR reveals Srp-binding sites in the CRMs of *LanB1* and *LanB2*. (**A**,**C**) Schematic representation of (A) *LanB1* and (C) *LanB2* transcripts, derived reporter constructs (green) and predicted GATA/Srp-binding sites with amplified regions beneath (yellow boxes with lowercase letters). The higher boxes indicate additionally amplified control regions outside of the displayed genomic loci. (**B**,**D**) qPCR amplified DNA of ChIP experiments with *srp::GFP* (green) and mock control (*white*^*1118*^, gray) genotypes illustrated as percentage of input. Samples are generated as independent biological replicates (n = 3). Welch’s two-sample t-test with adjusted p-values, ***p < 0.001, **p = 0.005, non-significant (n.s.) = p > 0.05. Mean ± SEM.

The experiments described above indicate a potential regulatory role of Srp for *LanB1* and *LanB2* expression. In order to test a direct binding ability of Srp to *LanB1*/*LanB2*, we performed ChIP analysis followed by subsequent qPCR. For *LanB1* expression, we analyzed the Srp enrichment at five genomic regions, with seven predicted GATA/Srp-binding sites (Supplementary Fig. S1, with marked genomic location). At the locus, represented by the *LanB1 IE 5*′ construct, we checked two GATA-transcription factor binding sites (GATA-TFBS 1 and GATA-TFBS 2) for enrichment. The results showed only a slight increase of Srp compared to the control (Fig. 4B; GATA-TFBS 1: control 0.05% of input (referred hereafter as %), Srp::GFP 0.08%; GATA-TFBS 2: control 0.03%, Srp::GFP 0.14%), whereas the enrichment of Srp at the locus represented by *LanB1 IE 3*′ was considerably higher than that in the control (Fig. 4B Srp-TFBS 1 + 2: control 0.03%, Srp::GFP 0.36%; GATA-TFBS 3 + 4: control 0.02%, Srp::GFP 0.32%; Srp-TFBS 3: control 0.03%, Srp::GFP 0.36%). The negative controls demonstrated that control primers amplify ChIP-DNA with a slight variation, but do not show enrichment over input (Fig. 4B control 1: control 0.06%, Srp::GFP 0.08%; control 2: control 0.05%, Srp::GFP 0.07%). Taken together, these results display a weak enrichment of Srp for the GATA-TFBS 2, located at *LanB1 IE 5*′, and a high significant enrichment of Srp in the *LanB1 IE 3*′ region. This was also evident in the context of reporter gene expression of these constructs (Fig. 1), because *LanB1 IE 5*′ display Srp-regulated *Laminin* expression only in the amnioserosa (Fig. 1C,F). This tissue persists just in the first half of embryogenesis and undergoes programmed cell death thereafter^25^. Therefore, just a small fraction of cross-linked cells could influence the enrichment of Srp in this approach. In contrast, *LanB1 IE 3*′ showed a high Srp enrichment and strong reporter gene expression in the endoderm, hemocytes and fat body, whose determination and differentiation depends on Srp activation (Figs 1 and 3).

For analysis of *LanB2* regulation, we investigated three genomic loci, including four potential GATA- and Srp-binding sites (Supplementary Fig. S2, with marked genomic location). The first Srp-binding site is located in the upstream enhancer (Srp-TFBS 1), one is found in the 5′ region of the intronic enhancer (GATA-TFBS 1), and two potential binding sites are located in the 3′ region of the intronic enhancer (Srp-TFBS 2 and GATA-TFBS 2; Fig. 4C). The ChIP analysis detected no enrichment of Srp in the upstream enhancer (Srp-TFBS 1: control 0.06%, Srp::GFP 0.06%), whereas both loci in the intronic enhancer showed a significant enrichment of Srp protein (GATA-TFBS 1: control 0.02%, Srp::GFP 0.15% for *LanB2 IE 5*′; Srp-TFBS 2 + GATA-TFBS 2: control 0.03%, Srp::GFP 0.20% for *LanB2 IE 3*′). These data are in agreement with the previous reporter gene analysis (Fig. 2), as we did not find any reporter gene expression of *LanB2 UE* in the embryo, while *LanB2 IE 5*′ and *LanB2 IE 3*′ displayed Srp-dependent Laminin expression patterns (Fig. 2).

Taken together, the strong Srp enrichment in both regions of *LanB2* intronic enhancer constructs and the strong enrichment found in *LanB1 IE 3*′ indicate that hemocyte and fat body regulation show the strongest effect for Srp binding. In addition, the enrichment in the *LanB1 IE 5*′ region indicates a regulatory role of Srp for Laminin expression in the amnioserosa.

### Ectopically expressed *srp* activates *Laminin* reporter gene expression

**Figure 5:**
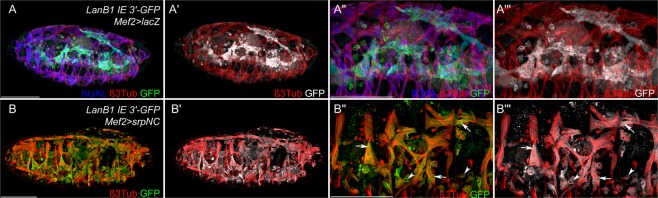
Myogenic *srp* expression leads to ectopic *Laminin* reporter activation and disrupted myogenesis. **(A**,A″′) Control animals show GFP reporter gene expression of *LanB1 IE 3*′ (**A**,A″ in green, A′,A″′ white) and *Mef2*-GAL4 driven *UAS*-*lacZ* activity (**A**,A″ blue) with anti-ß3Tubulin as muscles marker (red). (**B**,B″′**)** Ectopic expression of *UAS*-*srpNC* with *Mef2*-GAL4 is sufficient for *Laminin* reporter gene activation in somatic muscles (arrows) and unfused myoblasts (arrowheads), leading to disrupted myogenesis. GFP reporter gene expression (**B**,B″ green; B′,B″′ white) and anti-ß3Tubulin (red). Scale bars = 100 µm.

To test whether Srp is able to induce *LanB1* and *LanB2* expression, we expressed *srp*^27^ ectopically in all muscle cells using the *mef2-GAL4* driver in the background of our *LanB1 IE 3*′ (Fig. 5) and *LanB2 IE 3*′ (Supplementary Fig. S3) constructs, which displayed no myogenic expression (Figs 1J,J′ and 2J,J′). At the end of embryogenesis, the somatic musculature builds a specific stereotypical pattern. These myotubes were characterized by persistent *mef2* expression (revealed by *mef2-GAL4* driven *UAS-lacZ*, Fig. 5A,A″) and a characteristic cell shape in control animals (Fig. 5A,A″′). *LanB1 IE 3*′ GFP reporter gene expression in control embryos was restricted to fat body and hemocytes, whereas ectopically expressed *srp* induced reporter activity was detected in several somatic muscles (arrows, Fig. 5B″,B″′) and unfused myoblasts (Fig. 5B″,B″′), displaying ectopic activation of CRMs from *LanB1 IE3*′. Similar results were obtained with the *LanB2 IE 3*′ GFP reporter (Supplementary Fig. S3).

This gain-of-function experiments demonstrated that *srp* expression is sufficient for *LanB1* and *LanB2* enhancer activation. Interestingly, ectopic expression of *srp* led to a massive disruption of myoblast fusion, reminiscent of several myoblast fusion mutants^28–31^. So even at the end of morphogenesis, unfused myoblasts and disarranged fused muscles were clearly visible all over the somatic and the visceral mesoderm (Fig. 5B,B″′), indicating an important role of tissue-specific Srp expression and subsequent gene regulation.

## Discussion

Laminins are crucial components for BM construction. Our results indicate a common tissue-specific expression of LanB1 and LanB2. We also identified cis-regulatory modules (CRMs) for this tissue-specific expression. The expression of the Laminin β/γ subunits is essential for subsequent di- and trimerization, and reflects the onset of earliest ECM assembly.

Laminins are expressed in all germ layers (this study^7,8,15,16^) and an initial Laminin network exists prior to further ECM component secretion (this study^32^), which is responsible for complex developmental processes such as cell migration and organogenesis^7,8,33,34^, suggesting complex regulatory mechanisms. While the loss of Laminin reporter gene expression in *srp* mutants (Fig. 3) could also be explained by lack of differentiation of these tissues, ectopic activation of Srp in muscle cells (Fig. 5) indicates a regulatory role for Laminin expression. Based on the identified CRMs (Fig. 4), our results indicate that Srp the main GATA-transcription factor in *Drosophila*, act as Laminin regulator in distinct tissues and germ layers (Figs 1, 2, 5). In this context, it seems evident that Srp, owing to its expression in multiple tissues, is also able to regulate Laminin expression in a variety of tissues.

While, in *Drosophila*, Srp is the single most important GATA-factor for development, in vertebrates, multiple GATA-orthologues play important and tissue-specific roles during differentiation^24^. In this context, it seems interesting that Srp binds at the predicted GATA-TFBS (b in Fig. 4B,D), as this binding site was found with a PWM for mouse GATA1. The role of Srp for hemocyte/blood cell differentiation may be conserved throughout vertebrate development^35^. Therefore, it appears that there is a Srp-binding site closely related to the vertebrate GATA1-TFBS, while the Srp-TFBS reported here is similar to the vertebrate GATA4- and GATA6-TFBS^17^.

In cultured mouse embryonic stem cells, forced vertebrate GATA4 and GATA6 expression strongly enhances and silences laminin-1 production, respectively^36,37^. These experiments reveal no direct regulatory role of GATA-factors for Laminin regulation, but in the context of this study, it would be interesting to investigate, which GATA-factor takes over the regulatory role of Srp in vertebrates. Interestingly, the reciprocal loss of GATA4 or GATA6 in epithelium-derived tumor cells reveals a subsequent loss of Laminin expression^38^. In the context of our results and with the assumption that GATA-factors in vertebrates take over the role of Laminin regulation, it makes sense that epithelial cells show a strong expression of GATA-factors.

A recent study supports this notion, revealing a common role of Srp and human GATA4/6 in epithelial-mesenchymal-transition (EMT)^39^. Although, functions in epithelial maintenance and EMT seems to contradict each other, Laminin activation is required in mesenchymal cells for the migration-related processes^8,40^. As the presence of Srp disturbs the epithelial character in *Drosophila*^39,41^, we assume that the initial Laminin activation by Srp is essentially required in all Srp-dependent tissues, and that subsequent Laminin regulation for epithelial tissue assembly and maintenance must be ensured by other GATA-factors. Maintenance of gene expression in the differentiated midgut epithelium has already been demonstrated for dGATAe^42^, along with the fact that amnioserosa differentiation is dependent on dGATAa/pannier^43,44^.

## Material and Methods

### Fly stocks and genetics

Flies were grown under standard conditions^45^ and crosses were performed at 25 °C or 29 °C (for *GAL4*/*UAS* experiments). Staging of embryos was done according to^46^. The following mutations and fly stocks were used in this study: as control or wild-type stocks we employed *white*^*1118*^ or balanced sibling embryos. We use *mef2-GAL4* (BDSC 27390), *UAS-mCherry*.*NLS* (BDSC 38425), *UAS-lacZ* (BDSC 1777), *srp*^*3*^ (BDSC 2485), *srp*^*neo45*^ (BDSC 59020), *srp::GFP* (VDRC 318053) and *UAS-srpNC*^27^.

### Fluorescence antibody staining

Antibody staining of *Drosophila* embryos was essentially performed as described in^47^. The following primary antibodies were used in their specified dilutions: mouse anti-Green fluorescent protein (GFP, 1:250, Roche Diagnostics), rabbit anti-Green fluorescent protein (GFP, 1:500, abcam), guinea-pig anti-β3tubulin (ß3Tub, 1:6000^48^), rabbit anti-LamininB1 (LanB1, 1:400^49^) and rabbit anti-LamininB2 (LanB2, 1:400^49^). Alexa Cy-coupled secondary antibodies were purchased from Dianova and Jackson ImmunoResearch, and Hoechst 55380 from Sigma Aldrich. Embryos were embedded in Fluoromount-G (Southern Biotech) before visualization under Leica TCS SP2 confocal microscope.

### Generation of transgenic GFP reporter flies

Genomic DNA from wildtype *Drosophila melanogaster* was used for PCR amplification of respective sequences (LanB1 IE 5′ forward: CGAGTACGGATTCCCCACTG AAG; LanB1 IE 5′ reverse: CCGGCACTAGAAATGTTCTGAAAC; LanB1 IE 3′ forward: CAGTGGTCAGTCGCGAGGAA LanB1 IE 3′ reverse: CGATAAGCCGCAGCTCCAAC; LanB2 UE forward: CACGGGAAATTAAATGACGCGCCAA; LanB2 UE reverse: AGTGAAACTCTGTACTCTGCGCTCA; LanB2 IE 5′ forward: CGCAAGTACTCCAACCAGATCCGAC; LanB2 IE 5′ reverse: CTGATA CTGGAACTTACACCCCGCG; LanB2 IE 3′ forward: GAATGTGGTGTGTGTATGTGTGCGC; LanB2 IE 3′ reverse: GGTTGTACTTTGGTGTCTCGCTCG). The PCR fragments were sequenced, cloned into pGEM T-Easy vector (Promega) and subcloned into pH-Pelican-GFP vector^50^. Standard P-element transformation into *w*^*1118*^ embryos was performed by BestGene Inc. and the Renkawitz-Pohl lab.

### Bioinformatics

Evolutionary conservation in non-coding regions of *LanB1* and *LanB2* were identified by using PhastCons datasets of 14 insect species^19^. The intergenic regions upstream of the first transcription start site and the intronic sequences of *LanB1* and *LanB2* were analyzed by CIS-BP Database (PWMs log Odds, Threshold: 0.8)^18^ and JASPAR (Threshold: 0.8)^17^ for potential TFBSs. Further manually separations were performed according to the conservation score^19^ in the UCSC genome browser^51^ using BDGP R5 dm3.

### Chromatin immunoprecipitation

For preparation of chromatin, crosslinked 0–24 h old embryos (*w*^*1118*^ and *srp::GFP*) were used. Embryos were collected and dechorionated as described in^45^. Fixation was performed as previously described^52^. For sonification embryos were treated with a Branson 250 with four bursts for 30 s and 20–40% power in 3 ml RIPA buffer and 0.5 ml acid-washed glass beads (Sigma, G8772). Chromatin purification was performed as in^53^. 1 ml chromatin extract were preabsorbed with 20 µl Protein A/G agarose beads (Merck, IP05), while anti-GFP (Abcam, ab290, 1:500) antibody was incubated with 20 µl agarose beads in 1 ml RIPA buffer at 4 °C for one hour with an overhead shaker. The preabsorbed chromatin extract was incubated with the antibody-coupled agarose beads over night at 4 °C with an overhead shaker. DNA was purified with spin columns and used as template for qPCR.

### Real-time PCR

ChIP samples and 1% input were used for one 15 µl PCR reaction. Analyses were performed using iTaq Universal SYBR Green Supermix (Biorad) on a CFX96 Real-Time PCR detection system (Biorad). The results were presented as percentage of input of precipitated chromatin. The following primers were used: LanB1-GATA-TFBS1 (forward: ACTCCTTCTCCCTGCCTATTCT, reverse: CGGATGCGAAGGAGTGGAAA); LanB1-GATA-TFBS2 (forward; TTGTGGCACACTGCCTCTTT, revers: GGCATTTGAAGCCCTTGCC); LanB1-Srp-TFBS1 + 2 (forward: AAAACTTGGGTTCTTATCTCACCG, reverse: TTCTTATGAAGATTTCGACGGG); LanB1-GATA-TFBS3 + 4 (forward: ACTGATAAAAACAGCGATCCAACG, reverse: CCGACTACTCTCAATATAAGGTCCC); LanB1-Srp-TFBS3 (forward: TGGTACGAGACGAAAATAAATCGG, reverse: GCTTCTATCATCACTGTAAACCGC); LanB1-control1 (forward: AATCAAGCAAGTGGGAGCGA, reverse: CCAGACTGACCGAGGTGTTC); LanB1-control2 (forward: ATGGCCCAACCCACTTTTCA, reverse: TGCTAATCGCGCACAAACAA); LanB2-Srp-TFBS1 (forward: ATGAAACCGAAAGTGCGGC, reverse: AGCTGGACTCTCTGCTCTACT); LanB2-GATA-TFBS1 (forward: TCGACTTGTTGTTGCTGCCT, reverse: CGGGAAACACTCCGTCACAT); LanB2-TFBS-GATA-TFBS2-Srp-TFBS2 (forward: TTTCGAACCGTAAAGAGCCCA, reverse: AAGTCCTATGTTTATCAATGGCACC); LanB2-control1(forward: TCATTGTGGCGGTTTCCTGT, reverse: GCCTGATCCTTCTTGCTGGT); LanB2-control2 (forward: ATGAATTTGGAAGCGTGGCG, reverse: CGCCTGTAGCCCGGATAAAA).

### Statistical analysis

R 3.5.1 was used for statistical analysis^54^.

## Supplementary information

Dataset 1

## Data Availability

All data generated or analyzed during this study are included in the manuscript.
