# Supplementary material for: Serpent/dGATAb regulates Laminin B1 and Laminin B2 expression during Drosophila embryogenesis
Source: Sci Rep. 2019 Nov 4;9:15910. doi: 10.1038/s41598-019-52210-9 (PMC6828711; doi:10.1038/s41598-019-52210-9)
Supplement: Supplementary file 1 — Dataset 1 [file 41598_2019_52210_MOESM1_ESM.pdf]

Serpent/dGATAb regulates *Laminin B1* and *Laminin B2* expression during *Drosophila* embryogenesis

Uwe Toepfer[1, 2] (ORCID: 0000-0003-0278-6580), Maik C. Bischoff[1, 3] (ORCID: 0000-0002-6995-8159), Marek Bartkuhn[4] (ORCID: 0000-0001-6872-9082), and Anne Holz[1, #] (ORCID: 0000-0002-7028-6548)

[1] Justus-Liebig-Universitaet Giessen, Institut fuer Allgemeine und Spezielle Zoologie, Allgemeine Zoologie und Entwicklungsbiologie, Stephanstrasse 24, 35390 Giessen, Germany.

[2] Present address: Technische Universitaet Dresden, Institut fuer Genetik, Zellescher Weg 20b, 01217 Dresden, Germany.

[3] Present address: Philipps-Universitaet Marburg, AG Entwicklungsbiologie der Tiere, Karl-von-Frisch-Strasse 8, 35043 Marburg, Germany.

[4] Justus-Liebig-Universitaet Giessen, Institut fuer Genetik, Heinrich-Buff-Ring 58-62, 35392 Giessen, Germany.

# Correspondence should be send to: anne.holz@allzool.bio.uni-giessen.de

Supplementary Table, Figures and Figure Legends

Supplementary Table S1: GFP-Expression in *LanB1*/*LanB2* reporter constructs compared to previously published *LanB1* and *LanB2 mRNA* expression.

**Tissue**	***LanB1 mRNA***	**LanB1 5´-GFP**	**LanB1 3´-GFP**	***LanB2 mRNA***	**LanB2 5´-GFP**	**LanB2 3´-GFP**
Yolk sac			X	X		X
Early mesoderm	X	X		X	X	
Amnioserosa		X			X	
Hemocytes	X		X	X	X	X
Fat body	X		X	X	X	X
Somatic muscles	X	X		X	X	
Visceral muscles (fore- and hindgut)	X	X		X		X
Visceral muscles (midgut)	X	X		X		X
AMP and PMG	X		X	X	X	
Dorsal median cells	X		X	X		X
Salivary Glands/ Malpighian Tubules			X<br>X			

LanB1 and *LanB2 mRNA* expression depends on Montell and Goodman (1989), while *LanB2 mRNA* distribution is also derived from Wolfstetter and Holz (2012).

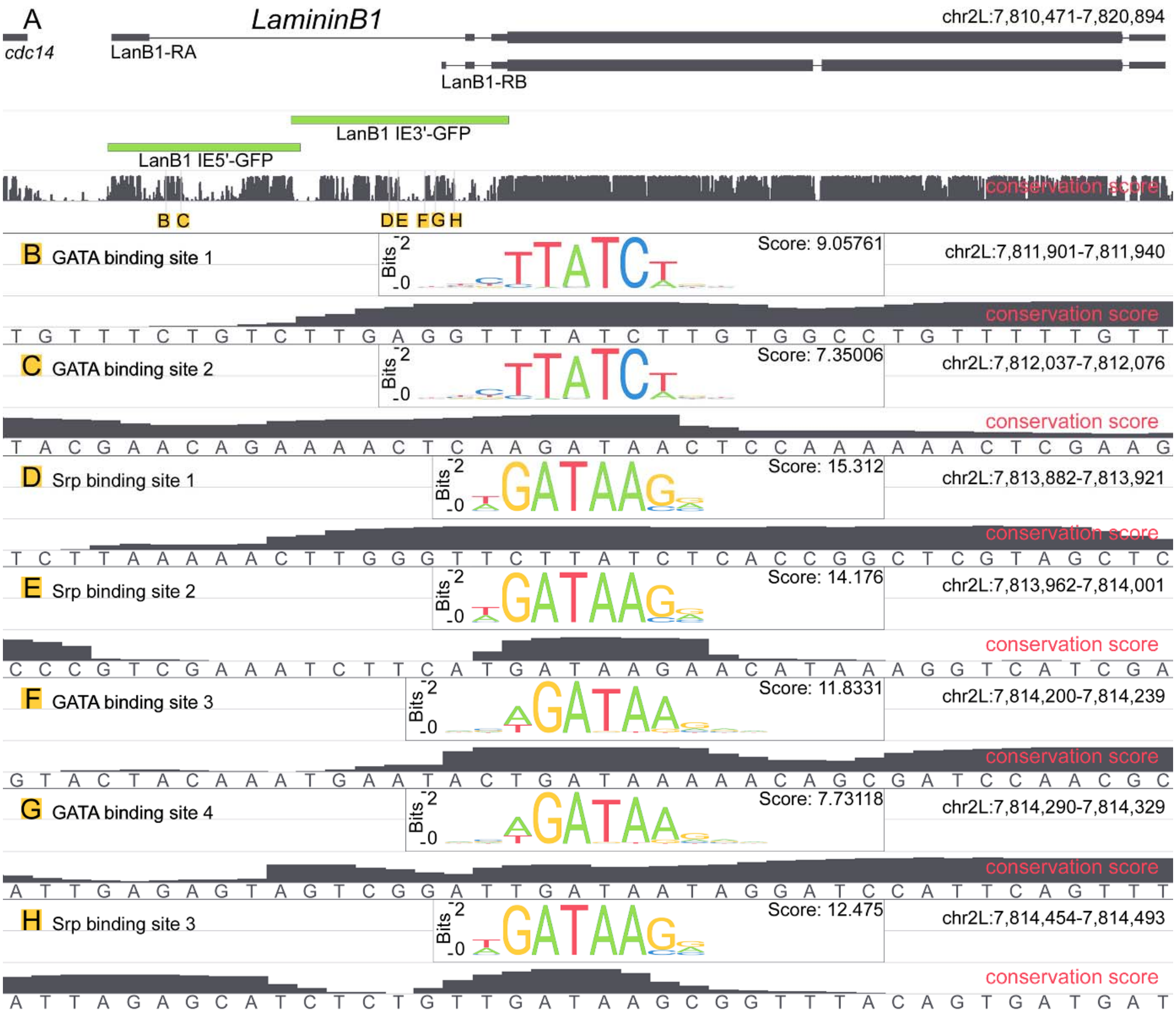

Supplementary Figure S1: Gene locus of *LanB1* with reporter gene constructs and predicted TFBSs. (A) Schematic representation of *LanB1* transcripts, derived reporter gene constructs (green) and conservation score (red) of the genomic region with predicted TFBSs. **(B-H)** Predicted TFBSs of either GATA- or Srp-binding sites with position frequency matrices (colored letters) and enrichment scores (boxes), conservation scores and accompanying DNA sequence.

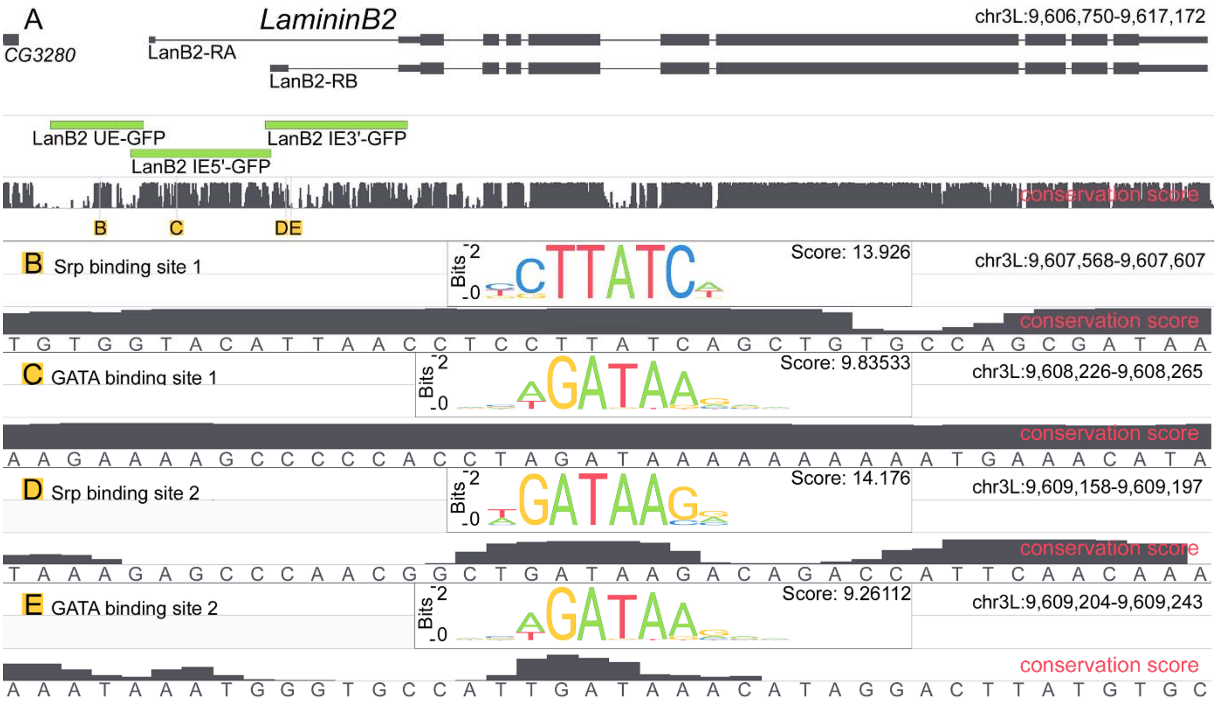

Supplementary Figure S2: Gene locus of *LanB2* with reporter gene constructs and predicted TFBSs. (A) Schematic representation of *LanB2* transcripts, derived reporter gene constructs (green) and conservation score (red) of the genomic region with predicted TFBSs. **(B-E)** Predicted TFBSs of either GATA- or Srp-binding sites with position frequency matrices (colored letters) and enrichment scores (boxes), conservation scores and accompanying DNA sequence.

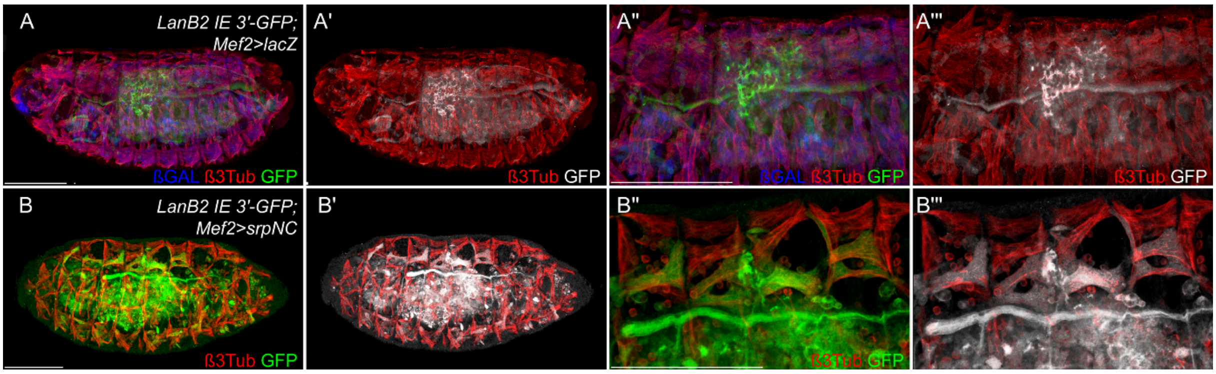

Supplementary Figure S3: Myogenic *srp* expression leads to ectopic *Laminin B2* reporter activation and disrupted myogenesis. (A-A´´´) Control animals show GFP-reporter gene expression of *LanB2 IE 3´* (A, A´´ in green, A´, A´´´ white) and *Mef2*-GAL4 driven *UAS-lacZ* activity (A, A´´ blue), with anti-ß3Tubulin as muscle marker (red). **(B-B´´´)** Ectopic expression of *UAS-srpNC* with *Mef2*-GAL4 is sufficient for *Laminin* reporter gene activation in somatic muscles and unfused myoblasts, leading to disrupted myogenesis. GFP reporter gene expression (B, B´´ green; B´, B´´´ white) and anti-ß3Tubulin (red). Scale bars = 100 µm.
